# The neutrophil protein CD177 is a novel PDPN receptor that regulates human cancer-associated fibroblast physiology

**DOI:** 10.1371/journal.pone.0260800

**Published:** 2021-12-08

**Authors:** Jillian L. Astarita, Shilpa Keerthivasan, Bushra Husain, Yasin Şenbabaoğlu, Erik Verschueren, Sarah Gierke, Victoria C. Pham, Sean M. Peterson, Cecile Chalouni, Andrew A. Pierce, Jennie R. Lill, Lino C. Gonzalez, Nadia Martinez-Martin, Shannon J. Turley

**Affiliations:** 1 Department of Cancer Immunology, Genentech, South San Francisco, California, United States of America; 2 Department of Microchemistry, Proteomics and Lipidomics, Genentech, South San Francisco, California, United States of America; 3 Department of Bioinformatics and Computational Biology, Genentech, South San Francisco, California, United States of America; 4 Center for Advanced Light Microscopy, Genentech, South San Francisco, California, United States of America; 5 Department of Research Pathology, Genentech, South San Francisco, California, United States of America; Universita degli Studi di Padova, ITALY

## Abstract

The cancer-associated fibroblast (CAF) marker podoplanin (PDPN) is generally correlated with poor clinical outcomes in cancer patients and thus represents a promising therapeutic target. Despite its biomedical relevance, basic aspects of PDPN biology such as its cellular functions and cell surface ligands remain poorly uncharacterized, thus challenging drug development. Here, we utilize a high throughput platform to elucidate the PDPN cell surface interactome, and uncover the neutrophil protein CD177 as a new binding partner. Quantitative proteomics analysis of the CAF phosphoproteome reveals a role for PDPN in cell signaling, growth and actomyosin contractility, among other processes. Moreover, cellular assays demonstrate that CD177 is a functional antagonist, recapitulating the phenotype observed in PDPN-deficient CAFs. In sum, starting from the unbiased elucidation of the PDPN co-receptome, our work provides insights into PDPN functions and reveals the PDPN/CD177 axis as a possible modulator of fibroblast physiology in the tumor microenvironment.

## Introduction

Interactions between cell surface proteins on adjacent cells fundamentally regulate cellular responses during homeostasis and disease, and therefore treatments aimed at blocking or modulating these interactions have important implications in preventing cancer and other immune diseases, such as pathogen infection. The biomedical relevance of membrane protein interactomes is well illustrated by the tumor microenvironment, a rich mixture of tumor and non-malignant cells including immune cells, vascular cells, and cancer-associated fibroblasts (CAFs), which establish an intricate relationship and in turn impinge on anti-tumoral responses. In particular, CAFs, which represent the majority of the tumor stroma, establish a dynamic crosstalk with tumor cells that plays a critical role in regulating tumor growth and progression [1, 2]. Similarly, although far less understood, compelling evidence indicates that fibroblasts critically influence anti-tumor immune responses and responsiveness to immunotherapies through multiple interactions with tumor-infiltrating immune cells [3–5].

Notwithstanding the emerging role for CAFs in immunosuppression and resistance to immunotherapy, the downstream molecular circuitry responsible for crosstalk between CAFs and tumor-infiltrating immune cells remains poorly understood. This fragmented understanding is in large part due to the biochemical intractability of membrane-spanning proteins, including difficulties to express active proteins that contain the relevant posttranslational modifications, their low abundance and solubility, and the low affinity, transient interactions that often characterize protein interactions on the cell surface [6–8]. These challenges have limited our understanding of receptor-ligand interactions which underly the basic crosstalk between human CAFs and immune cells in the tumor microenvironment, and consequently, the development of improved therapeutics targeting these interactions.

Numerous reports have shown that podoplanin (PDPN, gp38, Aggrus, or T1α), a single transmembrane (STM)-containing receptor highly expressed by CAFs, is overexpressed in tumors and is associated with poor prognosis [9–13]. However, fundamental aspects of PDPN biology have remained largely uncharacterized, including its relevant binding partners and the function of PDPN in the tumor microenvironment. While PDPN has been reported to bind to CLEC-2, CD44, CCL21, CD9, and galectin-8 [10, 14], the effects on these interactions specifically in fibroblasts or in vivo have not been examined. Most studies examining the role of PDPN in cancer have focused on cancer cells, but some papers exploring fibroblast-specific PDPN effects have found that PDPN+ CAFs support tumor growth and immunosuppression in the tumor microenvironment [12, 13, 15, 16]. Additionally, we and others have reported that PDPN controls contractility of mouse fibroblasts and is modulated by the C-type lectin receptor CLEC-2 (*Clec1b*) [17, 18]. Still a comprehensive study of the molecular interactions that may regulate CAF PDPN functions on the plasma membrane has not been conducted.

In an effort to better understand PDPN function in human CAFs, we utilized a recently developed platform for receptor discovery and improved detection of the low affinity interactions that often characterize membrane proteins [19]. Our comprehensive evaluation of the single transmembrane interactome of PDPN uncovered the neutrophil marker CD177 as a novel PDPN binding partner and confirmed the interaction with CLEC-2 in humans. Furthermore, quantitative mass spectrometry-based profiling of the CAF proteome and phosphoproteome in response to CLEC-2 or CD177 stimulation revealed that PDPN impacts signaling transduction, influencing multiple cellular pathways that include cytoskeleton organization or cell cycle regulation. Functionally, our results demonstrate that PDPN is a master regulator of CAF growth and actomyosin contractility, a function that is inhibited upon engagement by the novel binding partner CD177, as well as CLEC-2.

In sum, by examining the single transmembrane interactome, we identify the neutrophil protein CD177 as a novel negative regulator of the mechanoreceptor PDPN. These findings suggest a previously unappreciated crosstalk between CAFs and tumor-infiltrating immune cells that may regulate fibroblast physiology, ultimately suggesting new therapeutic avenues.

## Materials and methods

### Cell culture

The HEK cells used for protein binding analysis by flow cytometry were obtained from ATCC and grown in DMEM high glucose medium supplemented with 10% FBS, glutamine and antibiotics. The conditioned media library was produced in Expi293F cells (Thermo-Fisher). Cells were cultivated under the following conditions: 37°C, 8% CO2, 80% humidity and 150 rpm agitation speed. The primary human colorectal CAFs were purchased from Vitro Biopharma and grown in MSC-Gro low serum growth media (Vitro Biopharma). The primary human fibroblasts from bladder (primary normal bladder fibroblast cells, PCS-420-013) and colon (CCD-18Co, CRL-1459) were purchased from ATCC (Manassa, VA). The ovary fibroblasts (HOFs, #7336) were purchased from ScienCell Research Laboratories (Carlsbad, CA). All fibroblasts were cultured as recommended by the manufacturer and were used for a maximum of 6 passages.

### Cloning, protein purification, and biotinylation

For the generation of the recombinant proteins used in this study, the DNA sequences encoding the ectodomains were synthesized by GeneArt (Life Technologies) and cloned into a pRK5 vector (Genentech) containing a C-terminal human Fc tag (hIgG1). PDPN was expressed as an AviFc-tagged product with a TEV cleavage sequence to allow for removal of the Fc tag and generation of monomeric Avi-tagged protein when necessary. The proteins were expressed in mammalian cells, and subsequently purified using standard protocols [20]. Proteins showing less than 5% aggregation via size exclusion chromatography were dialyzed into PBS. Protein purity and identify were assessed by SDS-PAGE and mass spectrometry analysis. After removal of the Fc tag, the Avi-tagged product was site-specifically biotinylated *in vitro* using the BirA enzyme, following standard protocols [21]. CLEC-2 and CD177 proteins were produced in house and purified using standard affinity purification procedures. The proteins, expressed as recombinant ectodomains, were expressed in mammalian cells as Fc-tagged proteins. All other recombinant proteins were purchased from R&D, unless indicated.

### Conditioned media AlphaScreen platform

PDPN was screened against a library of human STM proteins using the Conditioned Media AlphaScreen technology, a platform based on the Alpha beads technology that was optimized for high throughput receptor-ligand discovery [19]. Screens were performed following the same experimental procedures previously described, without any further modification. Briefly, the library of STM protein ectodomains was prepared using high throughput, small-scale 1 ml transfections [19]. The conditioned media containing the STM proteins was harvested seven days post-transfection and incubated with the protein A-coated acceptor beads (5 mg/ml, Perkin Elmer #6760137R) for 1 hour at room temperature. Following incubation, the beads were pelleted using a microplate centrifuge (Beckman), washed with PBS-2% BSA and transferred to 384-well microplates (Optiplate, Perkin Elmer). In parallel, the purified, biotinylated PDPN ectodomain was incubated with the streptavidin-coated donor beads (5 mg/ml, Perkin Elmer #6760002) for 1 hour, washed and resuspended in PBS-2% BSA. Following incubation and washes, the donor beads/query protein were dispensed to 384-well plates containing the acceptor beads/STM library using a Tempest liquid handler (Formulatrix). Finally, the plates containing the microbeads mixtures were read using an Envision plate reader (Perkin Elmer) using manufacturer’s designed settings (excitation 680 nm; emission 520–620 nm). All procedures were performed using automated liquid handling devices to increase throughput and increase data quality.

### Surface plasmon resonance (SPR)

The interactions between PDPN, CLEC-2, and CD177 proteins were validated by SPR using the Proteon XPR36 Instrument (Biorad) or a Biacore 3000 (GE Healthcare). The purified proteins (CLEC-2 or CD177, expressed as recombinant ectodomains) were immobilized on GLC or CM5 sensor chips, respectively, using the amine coupling method. Avi-Fc-tagged PDPN was run as a soluble analyte at the indicated concentrations in PBS containing 0.01% Tween-20 (when the Proteon Instrument was used), or HBS-P buffer (0.01M Hepes, 0.15 M NaCl, 0.005% surfactant P20, pH7.4), for Biacore assays. Bulk refractive index changes were removed by subtracting a reference flow response. For K_D_ calculations, 300–400 resonance units were immobilized, and kinetic data were fit to equilibrium or Langmuir model to calculate kinetic parameters for CD177 and CLEC-2 binding, respectively. For kinetics calculations, Avi-tagged monomeric PDPN ectodomain was used as analyte. All sensograms were analyzed with BiaEvaluation 4.1 (Biacore) or Proteon Manager 3.1.0.6 (Proteon) software.

### Human tissue processing

Fresh human CRC samples digested using a previously published protocol [22]. Briefly, CRC samples were fragmented into small pieces (around 1 mm^3^) and digested in CO_2_-independent medium (Gibco, #18045–054) supplemented with 5% fetal bovine serum (FBS, PAA, #A11-151), 2 mg/ml collagenase I (Sigma-Aldrich, #C0130), 2 mg/ml hyaluronidase (Sigma-Aldrich, #H3506) and 25 mg/ml DNase I (Roche, #11284932001) for 45 min at 37°C with shaking (180–200 rpm). After tissue digestion, cells were filtered using a cell strainer (40 μm, Fisher Scientific, #223635447) and resuspended in PBS supplemented with 2 mM EDTA and 1% Human serum (Sigma P2918) to a final concentration at approximately 5x10^5^ cells in 50 μL.

### Flow cytometry

Once a single cell suspension was obtained, samples were incubated with human Fc block (Biolegend) for 10 min on ice, then washed and incubated with primary antibodies listed below for 20 minutes on ice. After a final wash, samples were filtered and analyzed on a Fortessa or Symphony machine (BD Biosciences). Data were analyzed on FlowJo (Version X, Tree Star). For FACS analysis of human tissue samples, the following antibodies were used: CD8 (BD Biosciences, clone SK1), CD4 (BD Bioscience, clone RPA-T4), CD11b (Biolegend, clone ICRF44), CD45 (Biolegend, clone 2D1), PDPN (eBioscience, clone NZ1.3), CD31 (BD Biosciences, clone WM59), and EpCAM (Biolegend, clone 9C4).

To measure the levels of PDPN on CAFs and fibroblasts in vitro, cells were collected and stained with 7-AAD and anti-PDPN (eBioscience, clone NZ1.3) for 20 minutes on ice. They were analyzed on a BD Fortessa cytometer.

### Generation of PDPN KO CAFs

The WT CRC CAFs were nucleofected with Cas9 protein complexed to guides targeting PDPN. Briefly, Alt-R crRNA and Alt-tracrRNA-ATTO550 (IDTd) was reconstituted to 100 μM with Nuclease-Free Duplex Buffer (IDT). The oligos were mixed at equimolar concentrations and annealed at 95°C for 5 minutes. Then, the annealed guides and TrueCut Cas9 Protein v2 (Thermo Fisher Scientific) were mixed at a molar ratio of 1:1.2 and incubated for 10 minutes at room temperature. Then 1 million CAFs were resuspended in 100 μL of solution R, mixed with the RNP, and nucleofected on a Lonza nucleofector 2b device following the manufacturer’s protocol for BJ cells (kit R, code X-001, Lonza). The cells were quickly rescued into 2 mL of media and allowed to recover for 1–2 days. The cells were expanded and then PDPN negative cells were sorted on an Aria (BD) to have a pure population of cells. Cells were expanded and banked to use in experiments. This process was completed using 2 different guides and four different independent batches of primary cells, with the same results.

### Cell confluency assay

WT or *PDPN*^-/-^ CAFs were plated at a density of 2,000 cells per well in a flat bottom 96 well plate. The cells were then placed in an Incucyte (Sartorius) and imaged every 4 hours for up to 200 hours. The confluence module in the Incucyte software was used to measure confluence over time in the phase images.

### Three-dimensional matrigel cultures

The effect of recombinant CLEC-2 or CD177 proteins or PDPN knockout on CAF elongation was analyzed essentially as described [18]. Briefly, 15,000 cells were resuspended in a matrigel-collagen mix, which was plated on glass-bottom 24-well plates. After incubation at 37°C for 20 minutes to allow the gels to set, 500 μL of media was added. Where indicated, 100 nM of CLEC-2 or CD177 was added to the media. The cells were cultured for two days before fixation with 4% PFA. The cells were stained with phalloidin-TRITC (Molecular Probes) and DAPI, and images were captured on a Leica SP5 using a HC PL APO 20x 0.70 NA CS objective, or a Nikon A1R confocal microscope dotted with a 20x 0.75NA Plan Apo VC air objective. The area and perimeter of the cells was measured in ImageJ (NIH) and a morphology index was calculated (perimeter^2^/ 4 × π × area).

### Gel contraction assays

To measure gel contraction, 100,000 CAFs were mixed with 50 μL of the same collagen-matrigel mix as above and plated in a 96 well plate. After the gels set for 20 minutes, a 200 μL pipette tip was used to separate the gel from the sides of the well and media was added. Gels were incubated for 48–72 h before imaging. Contraction was calculated by measuring the well diameter and the final gel diameter. For experiments with the recombinant proteins, they were added to the media each day during the experiment.

### Global phosphoproteomics

#### Proteomic sample preparation

CAFs were grown to ~70% confluency on 15 cm dishes. The day of the assay, the cells were starved for 2 hours, followed by stimulation with recombinant CLEC-2 or CD177 for 2 or 30 minutes. Stimulations were performed at 37°C in serum free media. After stimulation, the cells were washed with ice-cold PBS and subsequently harvested and lysed in 20 mM HEPES pH 8.0, 9 M urea containing 1 mM sodium orthovanadate, 2.5 mM sodium pyrophosphate, and 1 mM β-glycerophosphate. Lysates from 5 conditions were analyzed. These were: untreated, CLEC-2 treated for 2 min & 30 min, and CD177 treated for 2 min & 30 min. Two bioreplicates of each 5-plex experiment was performed and combined to make a 10-plex experiment. Samples were sonicated using a Misonix Microson XL sonicator followed by centrifugation at 20,000 g for 20 min at 15°C. Protein concentration was measured using Bradford assay (BioRad). Proteins (1 mg/condition) were reduced with 5 mM dithiothreitol (DTT) at 37°C for 1h followed by alkylation with 15 mM iodoacetamide (IAA) at room temperature for 20 min in the dark. Samples were diluted to a final concentration of 2M urea prior to digestion with Lys-C (Wako, Japan) at an enzyme:substrate ratio (E:S) of 1:50 at 37°C for 4 h followed by tryptic (Promega) digestion at an E:S ratio of 1:50 at 37°C overnight. The peptide solution was acidified with 20% trifluoroacetic acid (TFA) prior to solid-phase extraction using C18 cartridge (50 mg absorbent) from Waters. Peptides were eluted with 2 x 0.5 mL of 40% acetonitrile (ACN)/0.1% TFA followed by peptide concentration measurement using a quantitative colorimetric peptide assay kit (Thermo). Equal amounts per condition were subjected to lyophilization overnight.

#### TMT labeling

The peptide mixture was reconstituted in 1000 μL of HEPES (200 mM, pH 8.5) + 300 μL of ACN, and 100 μL of TMT reagent was added to each of the ten samples (each vial of TMT reagent (Thermo) was dissolved in 40 μL of ACN) (Table 1). Labeling was performed at RT for 1.5 h. A small portion (2 μL) from each condition was mixed, desalted, and analyzed to determine labeling efficiency as well as the relative protein abundance in each sample. The reaction was quenched with 100 μL of 5% hydroxylamine once labeling efficiency was determined to be at least 95%. Samples were mixed at equal amounts followed by acidification using 20% TFA and lyophilized overnight. The 10-plex TMT labeled peptide mixture was desalted using C18 cartridge (200 mg absorbent) from Waters. Sample was eluted with 3 x 1 mL of 60% ACN/0.1% TFA. A small amount (~0.5 mg) was saved for total protein profiling and ~6.5 mg was subjected to global phosphorylation analysis.

**Table 1:** List of TMT reagents and reporter ions used for each sample in two independent global phosphoproteome studies.

	Samples	TMT reagents	TMT reporter ions
**Bioreplicate 1**	DMSO	TMT10-126	126.1277
	Clec 2min	TMT10-127N	127.1246
	Clec 30 min	TMT10-127C	127.1309
	CD177 2min	TMT10-128N	128.1281
	CD177 30 min	TMT10-128C	128.1341
**Bioreplicate 2**	DMSO	TMT10-129N	129.1317
	Clec 2min	TMT10-129C	129.1376
	Clec 30 min	TMT10-130N	130.1348
	CD177 2min	TMT10-130C	130.1409
	CD177 30 min	TMT10-131	131.1381

#### Global phosphorylation & global protein analyses

Strong cation exchange (SCX) fractionation was performed using a PolySulfoethyl 4.6 mm ID x 200 mm, 5 μm, 200Å column (The Nest Group) at a flow rate of 1 mL/min on the HP1100 HPLC system (Agilent Technologies). Sixteen fractions were collected and then desalted followed by phosphopeptide enrichment using the TiO2 enrichment Phos-TiO kit (GL Sciences). For protein profiling, sample was subjected to high pH reverse phase fractionation where 96 fractions were collected and pooled into 24 fractions. Samples were desalted with C18 stagetip prior to mass spectrometry analysis.

#### Mass spectrometry analysis

Desalted peptides were reconstituted in 2% ACN/0.1% formic acid (FA)/water and loaded onto a C18 column (1.7 μm BEH, 130 Å, 0.1 x 250 mm, New Objective) using a NanoAcquity UPLC system (Waters) at a flow rate of 0.7 μL/min. A gradient of 2% to 30% solvent B (0.1% FA/2% water/ACN) at 0.5 μL/min was applied over 155 min with a total analysis time of 180 min to separate the peptides. Peptides were analyzed using an Orbitrap Fusion Lumos instrument (Thermo). Precursor ions (MS1) were analyzed in the Orbitrap (AGC target 1E6, 120,000 mass resolution, 50 ms maximum injection time) and 10 most abundant species were selected for fragmentation (MS2). Ions were filtered based on charge state ≥ 2 (z = 2, 3 & 4–6) and monoisotopic peak assignment and dynamic exclusion (45 s ± 10 ppm) was enabled. Each precursor was isolated at a mass width of 0.5 Th followed by fragmentation using collision-induced dissociation (CID at 35 NCE), MS2 AGC target was set at 2.0E4 with a maximum injection time of 200 ms. Multiple fragment ions were isolated using synchronous precursor selection (SPS) prior to HCD (55 NCE, SPS notches = 8, AGC target = 2.0E5, maximum injection time of 150 ms) MS3 fragmentation and Orbitrap analysis at 50,000 resolution. For global phosphorylation analysis, a neutral loss of 79.97 Da was specified to activate a multistage activation allowing for better identification of phosphorylated species. In addition, MS3 maximum injection time was set to 350 ms.

#### Mass spectrometry data analysis

MS/MS data was searched using the Mascot search algorithm (Matrix Sciences) against a concatenated forward-reverse target-decoy database (downloaded from UniProt in June 2016) consisting of Homo sapiens proteins and common contaminant sequences. Spectra were assigned using a precursor mass tolerance of 50 ppm and fragment ion tolerance of 0.8 Da. Static modifications included carbamidomethyl cysteine (+57.0215 Da), TMT (229.1629 Da) on both the N-terminus of the peptides and lysine residues. Variable modification included oxidized methionine (+15.994 Da) and phosphorylation on serine, threonine and tyrosine residues (+79.9663 Da) for phosphorylation analysis. Trypsin specificity with up to 3 missed cleavages was specified. Peptide spectral matches were filtered at 5% false discovery rate (FDR) followed by protein filtering at 2% FDR. For phosphorylated species, the AScore algorithm was applied to determine the exact phosphorylation site localization [23]. MS3 TMT quantification was performed using Mojave module, filtering out TMT peaks in MS3 scans that summed to less than 30,000 across all 10 channels. Each peptide was quantified by summing the TMT signal for each sample from all PSMs. Finally, peptides shorter than 7 residues were filtered out.

#### Mass spectrometry statistical analysis

For the global phospho-proteomics assay, all tryptic phospho-peptides covering identical phospho-site(s) were grouped under a single phospho-site group identifier, including groups that cover more than a single phospho-site. Then, for each phospho-site group (or protein in the global proteome assay) a model was fitted in MSstats v3.7.137 using a Tukey Median Polish summary on all quantified peptides across replicates with imputation of missing values below a censoring threshold of 28. Within MSstats, the model estimated fold change and statistical significance was computed for all compared treatment groups. Significantly altered phospho-site groups were determined by setting a threshold of |Log2(Fold-Change)| > 1 and p-value < 0.05. The union of all significantly altered phospho-site groups, compared to untreated, were then annotated and tested for over-represented biological annotations (Gene Ontology, PFAM, and KEGG), within each group comparison, with the GoStats package [24]. Significant annotations were defined by a q-value < 0.05, group size > 2 and genome occurrence < 1000 threshold. The significant terms were then further manually grouped into simplified, non-redundant categories for biological process terminology and tested for over-representation with a Fisher’s exact test. All proteomics raw data files have been deposited to the UCSD MassIVE database under the identifier MSV000083594 and data can be accessed using the login “MSV000083594_reviewer” and password "astaritapdpn".

## Results

### PDPN expressed by CAFs correlates with poor outcome in human colorectal cancer

**Fig 1:**
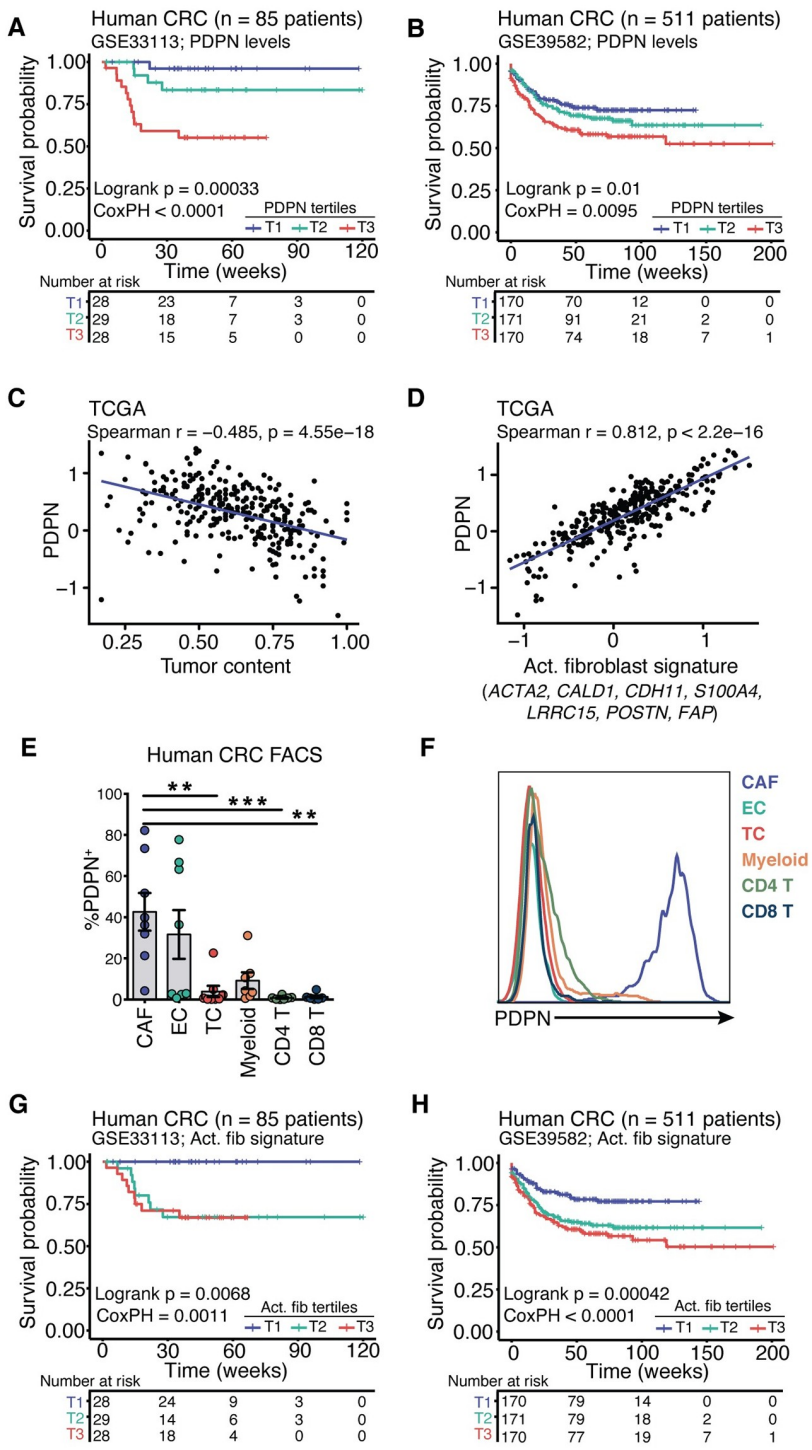
PDPN^+^ CAFs are associated with poor survival outcomes in CRC patients. Two publicly available CRC microarray gene expression datasets (GSE33113 and GSE39582) were analyzed to determine whether PDPN expression levels were significant prognostic factors in recurrence-free survival (RFS). **(A, B)** Kaplan-Meier curves for the survival probability of patients in GSE33113 (**A**) or GSE39582 (**B**) split into tertiles by levels of PDPN expression. Throughout the Fig., log-rank p-values are associated with Kaplan-Meier curves while Cox proportional hazard (CoxPH) p-values are associated with the univariate models detailed in Table 2. **(C)** The correlation between PDPN expression and tumor content. **(D)** The correlation between PDPN expression and a signature of activated fibroblasts. **(E, F)** Fresh human CRC tumors were digested and PDPN expression on major cell populations was analyzed by FACS. Bar graph indicating the percentage of each cell type that are PDPN^+^ **(E)** and a histogram of PDPN expression on each cell type **(F)**. **(G, H**) Kaplan-Meier curves for the survival probability of patients split into tertiles by expression levels of the activated fibroblast signature. See also S1 Fig and Table 2.

PDPN expression is highly elevated in several cancers, including squamous cell carcinoma of the head and neck, gliomas, and colon cancer and is associated with poor survival outcomes [9]. However, most studies are largely based on immunohistochemistry analysis and often do not define the cellular source of PDPN, which is important when considering the function of this protein as it can be expressed by tumor cells, CAFs, lymphatic endothelium, and some immune cells [2, 9]. We first sought to determine if PDPN expression was associated with clinical outcome, focusing on colorectal adenocarcinoma (CRC). To determine whether PDPN expression at the RNA level was a significant prognostic factor in CRC, bulk tumor gene expression and recurrence-free survival (RFS) data from two previously published human CRC studies were queried. In a cohort consisting of stage II patients (GSE33113, n = 85) [25] we found that PDPN expression was significantly associated with reduced survival (Fig 1A). Similarly, in a larger CRC cohort that included patients from all stages (GSE39582, n = 511) [26], PDPN expression was again significantly associated with reduced RFS, both in the entire cohort and in stage II patients (Fig 1B and S1A Fig). In late-stage disease, PDPN expression was a highly significant risk factor for stage IV (S1B Fig), but not for stage III CRC patients (Table 2).

**Table 2:** Recurrence-free survival (RFS) association of PDPN transcript and fibroblast signature levels in public CRC datasets (GSE33113 and GSE39582).

Gene or Signature	Survival Model	Dataset	Stage	N	HR	95% Confidence Interval	P-value	Signif. level
***PDPN***	***RFS ~ PDPN***	GSE33113	II	85	3.696	2.043–6.686	1.55E-05	***
	***RFS ~ PDPN***	GSE39582	I	37	0.6827	0.06547–7.119	7.50E-01	
			II	235	1.286	0.9968–1.658	5.30E-02	^
			III	183	0.956	0.7365–1.241	7.35E-01	
			IV	56	1.705	1.205–2.412	2.60E-03	***
			ALL	511	1.227	1.051–1.432	9.48E-03	**
***Activated Fibroblasts***	***RFS ~ Act*. *Fib***.	GSE33113	II	85	3.054	1.559–5.98	1.13E-03	**
	***RFS ~ Act*. *Fib***.	GSE39582	II	235	1.575	1.153–2.152	4.36E-03	**
			ALL	511	1.55	1.287–1.866	3.83E-06	***
	***RFS ~ Act*. *Fib*. *+ STAGE***	GSE39582	ALL	511	1.472	1.2161–1.781	7.20E-05	***
***FAP Fibroblasts***	***RFS ~ FAP Fib***.	GSE33113	II	85	4.055	1.694–9.703	1.66E-03	**
	***RFS ~ FAP Fib***.	GSE39582	II	235	1.618	1.129–2.317	8.74E-03	**
			ALL	511	1.612	1.303–1.994	1.07E-05	***
	***RFS ~ FAP Fib*. *+ STAGE***	GSE39582	ALL	511	1.516	1.2207–1.882	1.66E-04	***

Given that many cell types can express PDPN in the TME, we next asked whether PDPN expression was derived primarily from fibroblasts or tumor cells. To do so, a list of activated fibroblast genes from the literature and in-house data was curated and used to infer fibroblast levels from RNA-seq data. The genes are *ACTA2*, *CALD1*, *CDH11*, *FAP*, *LRRC15*, *PDPN*, *POSTN*, *S100A4*. DNA copy-number based tumor content data [27] was utilized to represent tumor cells in the sample. Interestingly, we found that *PDPN* mRNA levels were negatively correlated with tumor content (Fig 1C) and strongly correlated with inferred activated fibroblast levels (Fig 1D). These results suggest that PDPN expression in the TME is largely derived from fibroblasts. To confirm this result, we obtained human CRC tumors and analyzed PDPN expression on major cell types by FACS. An average of 40% of CAFs in each tumor were PDPN positive, and while lymphatic endothelial cells also express PDPN, CAFs had many more molecules per cell (Fig 1E and 1F, S1C Fig).

Next, to assess the significance of CAFs more deeply in human CRC, we generated a second gene signature representing CAFs in addition to the activated fibroblast one used above: a FAP^+^ pathogenic fibroblast signature. To do this, a differential expression test among four sorted cell populations (FAP^+^ fibroblasts, CD31^+^ endothelial cells, immune cells, and tumor cells) was performed [28], and the top 35 genes expressed only by fibroblasts and not by other cells were used to generate the signature (Table 3). We then investigated whether the activated fibroblast and FAP^+^ fibroblast gene signatures were prognostic in human CRC. Both signatures were significantly associated with reduced RFS in both GSE33113 and GSE39582, for the entire cohort as well as for stage II patients (Fig 1G and 1H, and S1D–S1G Fig). Interestingly, both fibroblast signatures remained significant predictors of RFS when included in multivariate models fit to all patients while controlling for stage (Table 2 and Supplemental information).

**Table 3:** Genes in FAP^+^ fibroblast signature and expression levels across sorted cell types.

					Average expression				
GeneSymbol	logFC	t	P.Value	adj.P.Val	EpCAM+	CD45+	FAP+	CD31+	all cells
LUM	7.19308115	35.3139128	1.70E-23	3.53E-19	1.40040376	1.60445265	8.71176024	1.55118084	3.31694937
CXCL14	6.03015105	24.0745447	2.27E-19	9.41E-16	2.33522259	2.04403339	8.23664139	2.24021503	3.7140281
AXL	4.51231586	20.4661023	1.17E-17	3.48E-14	2.41667865	2.55913915	6.9251358	2.26264202	3.5408989
VCAN	5.63154046	18.0147843	2.49E-16	4.69E-13	2.07637682	2.50051759	8.115062	2.87367019	3.89140665
SPON2	4.39501999	17.031275	9.37E-16	1.39E-12	2.56757098	2.9999312	7.12604115	2.62556131	3.82977616
FIBIN	4.00037496	16.8895736	1.14E-15	1.48E-12	2.29978418	2.23936459	6.20826318	2.0845159	3.20798196
CYGB	4.42690977	16.7824147	1.32E-15	1.53E-12	2.31706058	2.33546892	6.92222735	2.83342322	3.60204502
PCDH18	3.69897361	16.3403118	2.48E-15	2.70E-12	2.72655021	2.69110957	6.39751091	2.67795214	3.62328071
ISLR	4.9977407	15.095215	1.56E-14	1.41E-11	2.43522776	2.38090673	7.44790411	2.53435575	3.69959859
GPNMB	4.52944591	14.2335775	5.99E-14	4.97E-11	2.75280662	2.96190655	7.33947507	2.71537432	3.94239064
PDGFC	3.82419051	14.1391255	6.97E-14	5.56E-11	1.76362833	2.08393581	5.82924698	2.16760527	2.9611041
MEIS1	3.58955935	13.4291911	2.23E-13	1.60E-10	2.3780163	1.94108428	5.79055452	2.28388493	3.09838501
C4A /// C4B /// LOC100292046	3.31847486	13.0665004	4.13E-13	2.67E-10	2.39568223	2.5945314	5.88802473	2.71843598	3.39916858
ASPN	3.98565876	12.6555752	8.40E-13	4.84E-10	2.31628389	2.7249375	6.49181237	2.47723946	3.50256831
COL8A1	4.01803272	12.2920589	1.60E-12	8.28E-10	2.78259124	2.45244548	6.71367016	2.85187559	3.70014562
GREM1	3.88866254	12.1587295	2.03E-12	1.03E-09	1.68427858	1.47290709	5.46626306	1.57561589	2.54976615
MMP3	5.0885164	11.3810798	8.51E-12	3.75E-09	1.89457043	1.69268306	6.92613481	1.92560172	3.1097475
ADAMDEC1	4.70625553	11.0829627	1.50E-11	6.23E-09	1.11891724	1.43324281	5.95461976	1.19293263	2.42492811
MFAP4	3.15346624	10.4150649	5.56E-11	2.26E-08	1.89058524	1.871057	5.06999834	1.98795406	2.70489866
TWIST1	3.02315709	10.3499666	6.33E-11	2.53E-08	2.16581835	2.29543144	5.21756981	2.12198837	2.95020199
WNT2B	2.97709497	10.1845352	8.84E-11	3.34E-08	2.9371018	2.32028399	5.66784363	2.8148602	3.43502241
MXRA8	3.24208188	10.0450901	1.17E-10	4.20E-08	2.74274777	2.65946976	5.88807444	2.53576016	3.45651303
SDK1	3.05852336	10.050661	1.16E-10	4.20E-08	2.66183316	2.21512299	5.52975974	2.53675297	3.23586721
LRRN4CL	3.34797	9.83629266	1.81E-10	6.24E-08	2.10861241	1.5420821	5.19631839	1.89435068	2.6853409
WNT5A	4.19946715	9.60847443	2.91E-10	9.42E-08	1.07377208	1.08282947	5.28309242	1.09427425	2.13349206
FNDC1	3.3485901	9.59987577	2.96E-10	9.44E-08	2.60171628	2.58443732	5.96461573	2.6619233	3.45317316
CHI3L1	4.28455717	9.42977509	4.24E-10	1.31E-07	2.96362673	2.86166315	7.21226167	2.95782361	3.99884379
COL7A1	2.75433407	9.3378982	5.16E-10	1.53E-07	2.82929048	2.6164538	5.45382244	2.65272081	3.38807188
LOXL1	2.81018719	9.08959933	8.80E-10	2.53E-07	2.87091799	2.61912077	5.5218389	2.64491637	3.41419851
PLXDC1	2.91317831	8.58888266	2.65E-09	7.05E-07	2.45173707	2.91564477	5.45213455	2.24948688	3.26725082
COL11A1	3.42467138	8.02728745	9.49E-09	2.19E-06	1.65010987	1.7237981	5.12498818	1.72704242	2.55648464
TBX2	3.01723634	7.83839565	1.47E-08	3.18E-06	2.13947242	2.23206179	5.30973318	2.50595633	3.04680593
NOTCH3	2.36806199	6.16351089	8.75E-07	0.00013547	2.92654284	2.67265753	5.22744073	2.97893585	3.45139424
SLIT3	2.80558497	5.55647049	4.15E-06	0.00055838	2.16796754	2.21396634	5.00071286	2.2034498	2.89652413
SFRP4	2.75206796	4.73886214	3.49E-05	0.00369712	2.67369052	2.69774011	5.33240291	2.36957423	3.26835195

Average expression cutoffs were as follows: FAP+ > 5, EpCAM+ < 3, CD31+ < 3, CD45+ < 3. Adj. P Val <0.1 was used as the criterion to select the genes.

Taken together, these data indicate that not only is PDPN itself an indicator of poor outcome in human CRC, but specifically PDPN-expressing CAFs in the TME may also contribute to reduced survival.

### The neutrophil protein CD177 is a novel binding partner of PDPN

The functions of PDPN in human fibroblasts and the tumor microenvironment remain largely uncharacterized, in part due to a limited understanding of the landscape of PDPN co-receptors on the cell surface [9]. A number of extracellular binding partners have been reported for PDPN, including the cell surface-expressed proteins CLEC-2, CD44 and CD9, and the secreted factors CCL21 and galectin-8 [10, 14]. However, a systematic characterization of the landscape of PDPN interactors has not been performed, especially using techniques with improved sensitivity for detection of interactions between membrane proteins. Thus, To gain insights into the PDPN interactome on the cell surface, we applied a previously developed platform for high throughput receptor-ligand discovery, the Conditioned Media AlphaScreens [19]. In brief, this platform relies on a beads-based proximity method to query interactions between membrane protein ectodomains, immobilized on acceptor and donor beads, respectively. An interaction between two proteins brings the beads in proximity, triggering a series of energy transfer reactions that generate a chemiluminescence signal that can be measured using a plate reader. To enable an unbiased elucidation of binding partners on the cell surface, we engineered a library consisting of most STM human proteins, expressed as Fc-tagged proteins secreted to the conditioned media of transfected cells. We have previously demonstrated that the Conditioned Media AlphaScreen platform enables identification of diverse receptor interactomes, including those binding partners characterized by micromolar affinities [19], thus representing an important addition to the arsenal of receptor-ligand discovery tools.

**Fig 2:**
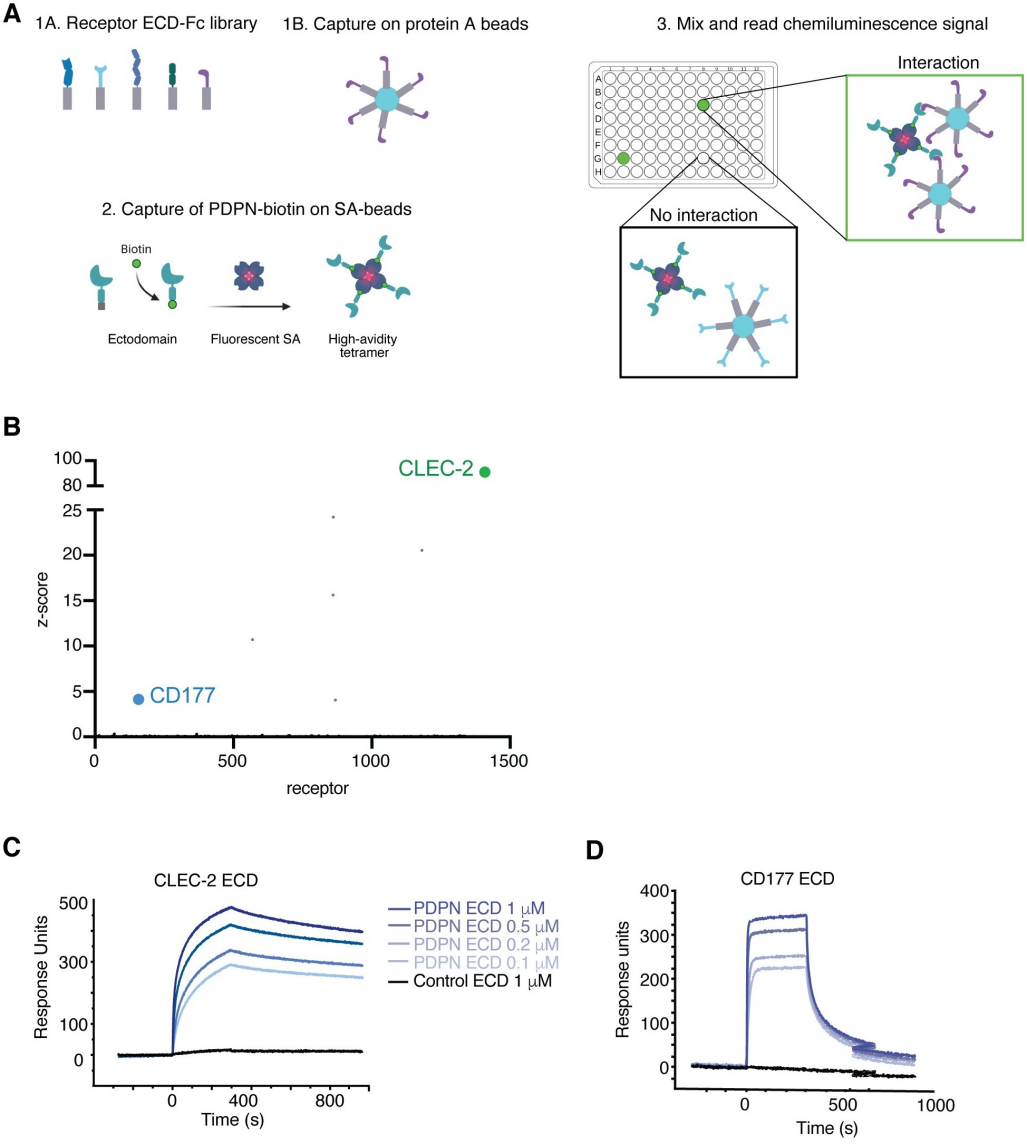
The PDPN cell surface interactome identifies the neutrophil marker CD177 as a novel binding partner. **(A)** Schematic representation of the Conditioned Media AlphaScreen platform for receptor-ligand discovery. A library consisting of 1,200 unique STM proteins as ectodomain-Fc fusions was expressed in the conditioned media of human cells. Conditioned media enriched in individual STM proteins was captured on acceptor protein A-coated beads. Site-directed biotinylated PDPN ectodomain was captured on streptavidin-coated donor beads, and subsequently incubated with the library of STM proteins captured on acceptor beads. Putative PDPN binding partners are detected by measuring the chemiluminescence signal. **(B)** PDPN screens identify CD177 as a specific, high-scoring hit. Hits shown in grey circles have been empirically determined as non-specific binders in previous screens. Analysis of the new interactions identified using SPR. Recombinant **(C)** CLEC-2 or **(D)** CD177 ectodomains were immobilized on sensor chips and purified PDPN, expressed as a Fc-tagged ectodomain, was injected at the concentrations indicated. Dissociation constant (K_D_) values for each interaction, measured using recombinant PDPN expressed as a monomeric ectodomain, are indicated below each plot.

To evaluate the cell surface interactome of PDPN using this platform, PDPN was expressed as a site-directed biotinylated recombinant ectodomain that was immobilized on streptavidin-coated donor beads (Fig 2A and Methods). Notably, when PDPN was screened against the STM protein collection, we identified a unique putative binding partner, CD177/NB1, a cell surface glycoprotein mainly expressed by neutrophils and a fraction of human intra-tumoral Tregs [29–31] (Fig 2B). Additional high scoring hits observed in the screens were previously identified as non-specific binding partners and therefore were not considered for further evaluation [19]. The known PDPN binding partner, CLEC-2 [32], not present in the library screened, was spiked-in as positive control (Fig 2B), further confirming this interaction in the human system.

Next, to confirm the newly identified interaction between PDPN and CD177 and further characterize it biochemically, PDPN binding to CLEC-2 and CD177 was probed using surface plasmon resonance (SPR) with recombinant, purified proteins. These studies corroborated the interaction between PDPN and CLEC-2 (Fig 2C), as well as PDPN and CD177 (Fig 2D). Interestingly, PDPN interacted with CD177 with micromolar binding affinity (K_D_ = 3.3 ± 0.7 μM), one order of magnitude lower relative to the binding affinity observed for CLEC-2 (K_D_ = 210 ± 0.3 nM), illustrating the sensitivity of our approach to detect interactions characterized by a range of affinities. Notably, while the CLEC-2-PDPN interaction is conserved in mouse, we were unable to detect any binding between mouse CD177 and mouse PDPN using multiple readouts (not shown), possibly due to the low sequence conservation in mouse and human CD177. These results are interesting as they suggest this interaction plays a function specific to the human immune system.

### The CAF phosphoproteome suggests that PDPN engagement controls multiple cellular functions

**Fig 3:**
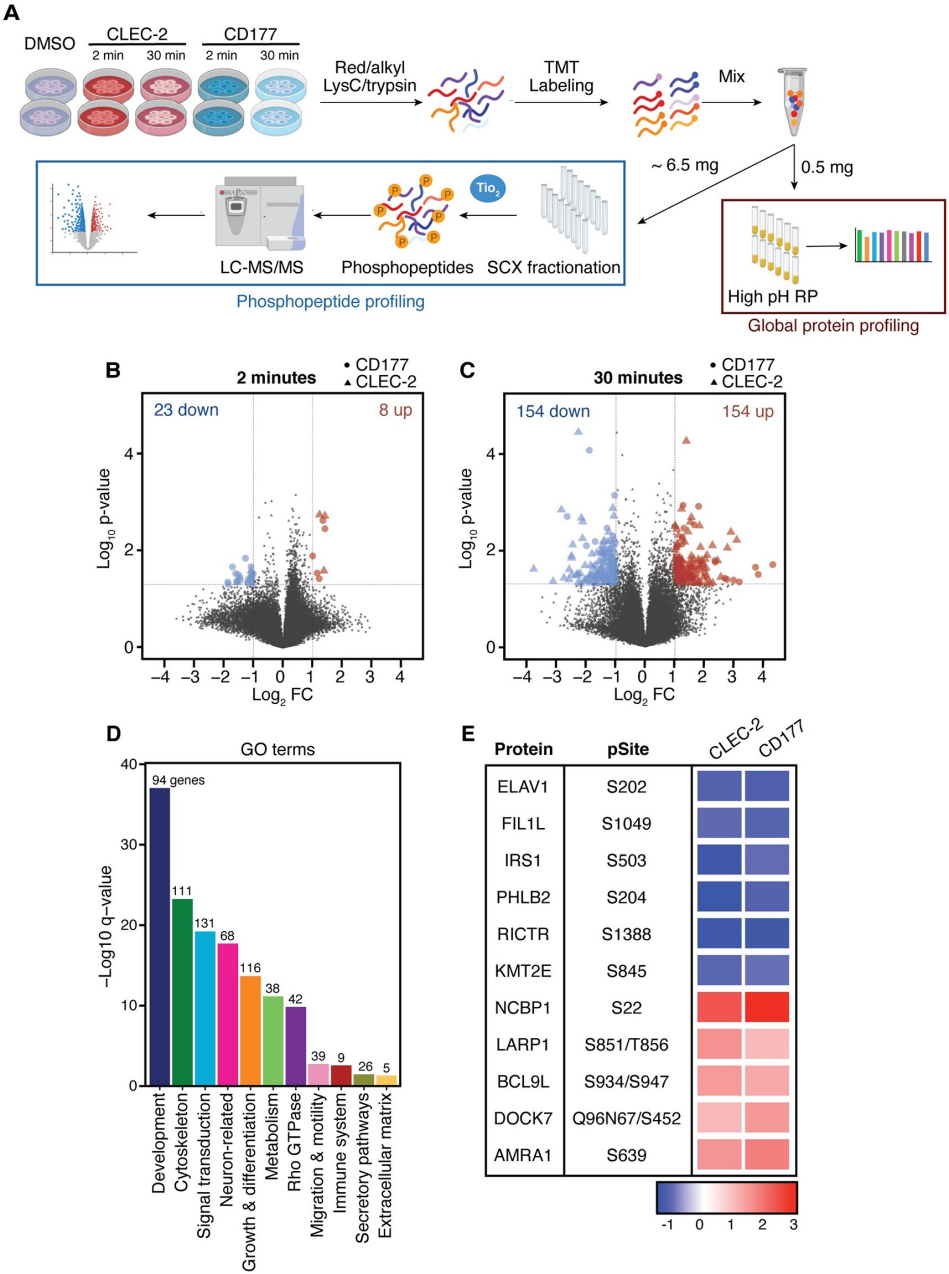
CLEC-2 and CD177 have a profound effect on the CAF phosphoproteome. **(A)** Schematic detailing the experimental workflow of multiplexed phospho- and global proteome profiling in parallel with TMT and fractionation for deep coverage. **(B-C)** Volcano plots depicting significant changes in CD177- (circle) and CLEC-2- (triangle) treated CAFs relative to untreated cells after **(B)** 2 min, or **(C)** 30 min of stimulation. Phosphosites that changed significantly (|Log2FC| > 1 and p < 0.05) in at least one treatment are represented. **(D)** Plot summarizing the enriched (q<0.05) GO pathways comprising proteins with significantly altered phosphosites compared to untreated cells. Numbers indicate the phosphosite groups associated with each curated GO term. **(E)** Heatmap representing the relative change across treatment conditions at 30 min of stimulation (relative to untreated cells) for selected phosphorylation sites on selected proteins.

Having validated the interaction between CD177 and PDPN, we next set out to study the downstream signaling outcomes of CD177 binding to PDPN. Primary human CAFs isolated from CRC tissue, which endogenously express high levels of PDPN, were utilized as a model cell line in order to recapitulate a physiologically relevant setting. Studies from tumor cells suggested that PDPN may play a role in migration, involving rapid signaling events. Thus, to comprehensively survey signaling pathways and effector proteins modulated by CD177 and CLEC-2, we performed global quantitation of the global proteome and phospho-proteome using a deep, multiplexed proteomics approach (Fig 3A). Human CRC CAFs were stimulated with recombinant CD177 or CLEC-2 for 2 or 30 minutes to capture quick signaling events. Then, a proteomics profiling method that combines 10-plex tandem mass tag (TMT) labeling of proteome fractions was used for relative quantitation of phosphopeptides across all conditions, followed by computational data processing for total protein, phosphosite quantitation, and finally statistical testing [23].

In the global phosphoproteome analysis, we detected 4,122 phosphosites, and for 70% of these we also detected the total protein. Given that we did not see changes in the global protein levels as a result of the CLEC-2 or CD177 stimulation (S2A Fig and S1 and S2 Tables), we can confidently conclude that changes observed in these phosphopeptides were not the result of changes in global protein levels, but rather changes in the phosphorylation status. In fact, PDPN engagement by CD177 and CLEC-2 substantially impacted the CAF phosphoproteome. Changes in the global phosphoproteome were more prominent after 30 min of stimulation compared with 2 min (Fig 3B and 3C and S1B Fig). At 2 min, around 30 phosphopeptides were significantly modulated by either CD177 or CLEC-2 (Fig 3B) whereas ~300 phosphosites, corresponding to 226 unique proteins, were significantly regulated (hyper- or hypo-phosphorylated) after 30 min of stimulation with the PDPN binders (Fig 3C and S1–S3 Tables).

To functionally categorize the pathways modulated by PDPN engagement, the phosphoproteins significantly regulated by CD177 or CLEC-2 were analyzed for enrichment in Gene Ontology (GO) terms. These results highlighted roles for PDPN in cytoskeleton rearrangement, cell motility, and extracellular matrix deposition, in line with previous data from our laboratory and others [17, 18, 33] (Fig 3D, S2B Fig, and S4–S6 Tables). Modulators of these processes were substantially altered upon PDPN targeting, including plectin (PLEC), the microtubule-associated proteins MAP1S or MAP4, the guanine-nucleotide exchange factors GBF1 and RGH10, or the pleckstrin homology-like domain family B members 1 and 2 (PHLB1 and PHLB2), among others (Fig 3E, S2B Fig, and S5 Table). Moreover, these results also suggested prominent alterations in proteins controlling other pathways, such as signaling transduction, vesicle trafficking or biosynthesis processes, as a result of CLEC-2 and CD177 binding. Interestingly, we also observed phosphorylation changes in a substantial number of proteins involved in nucleic acid metabolism and control of cell cycle, suggesting a previously unappreciated role for PDPN in cell growth and proliferation. For example, several transcriptional regulators and RNA binding molecules such as MED1, BCL9L, WWTR1/TAZ, LARP1, and NCBP1, alongside known regulators of proliferation such as filamin A interacting protein 1-like (FIL1PL1) or ELAV-like protein (ELAVL1) were significantly hyper- or hypo-phosphorylated upon PDPN engagement (Fig 3E and S2B Fig).

### PDPN is a main regulator of human CAF contraction and growth

Although in many cases the functions of these regulatory sites have not been elucidated, this global assessment of the phosphoproteome supported the idea that PDPN plays a role in modulating CAF physiology, including cell morphology, cytoskeleton rearrangement, or cell growth. To determine whether the changes in the phosphoproteome translated into true functional changes, we then examined the impact of PDPN expression and engagement by its ligands in CRC CAFs.

**Fig 4:**
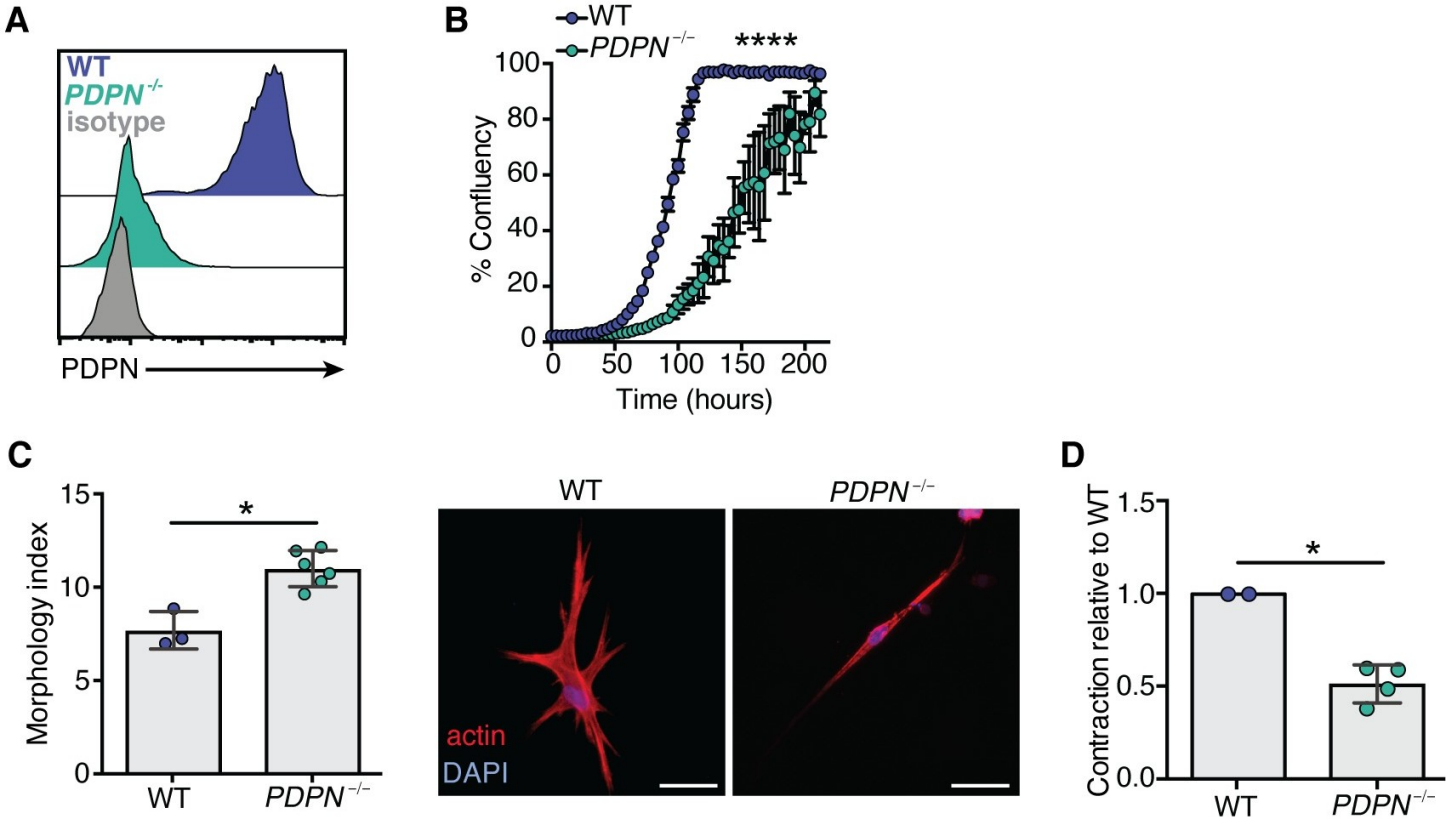
PDPN controls primary human CRC CAFs growth and contractility. **(A)** Histogram showing the expression of PDPN on WT CAFs and *PDPN*^-/-^ CAFs compared to an isotype control analyzed by FACS. **(B)** Graph depicting the percent confluency of WT and *PDPN*^-/-^ CAFs over time. Each point represents the mean of 16 different fields of view from 4 wells per condition, and the plot is representative of 4–6 independent experiments. ****p < 0.0001, ANOVA. **(C)** Left: Morphology index (perimeter^2^/4**area) of WT and *PDPN*^-/-^ CAFs seeded into 3D gels. Each dot represents an average of one well containing >50 cells and the plot is representative of 3 independent experiments. Right: Representative images of CAFs in 3D gels with staining for actin (red) and nuclei (blue). **(D)** Graph representing the relative contraction of *PDPN*^-/-^ cells compared with WT cells. Each dot represents the average of 3–4 wells each from 4 independent experiments. *p < 0.05, **p < 0.01, Mann Whitney.

First, to investigate the function of tonic PDPN signaling, we generated PDPN-deficient CAFs using the Cas9-RNP-CRISPR system and confirmed the loss of PDPN expression by FACS (Fig 4A). We examined whether the lack of PDPN impacted the growth rate of these cells, given that fibroblast contractility can control cell growth and survival. Notably, *PDPN*^*-/-*^ fibroblasts grew slower compared with *PDPN*^+/+^ cells (Fig 4B), as measured by percent confluence over time. Over the 200 hours of the assay, the PDPN-deficient CAFs never reached full confluence, while the WT cells did around 100 hours. These results are in line with our analysis of the phosphoproteome in CAFs and suggest that PDPN impinges on cell cycle pathways (Fig 3D). Next, we analyzed actomyosin contractility, a functional hallmark of myofibroblasts [17, 18]. Upon seeding these cells into 3D collagen matrices, we observed that *PDPN*^*-/-*^ CAFs exhibited a markedly different morphology compared to WT CAFs, with an approximately 70% more elongated phenotype (Fig 4C).

To test cell contraction, the CAFs were again seeded into collagen-based 3D matrices but this time allowed to contract the gels over a period of three days. Consistent with our hypothesis that PDPN regulates human fibroblast contractility, the *PDPN*-deficient cells were impaired in their ability to contract the collagen gels compared with WT cells (Fig 4D). Together, these results support the finding that PDPN signaling has an impact on a range of basic fibroblast functions, promoting actomyosin contractility in human fibroblasts and their growth in a cell-autonomous manner.

### CD177 inhibits PDPN function as a master regulator of cell contraction

**Fig 5:**
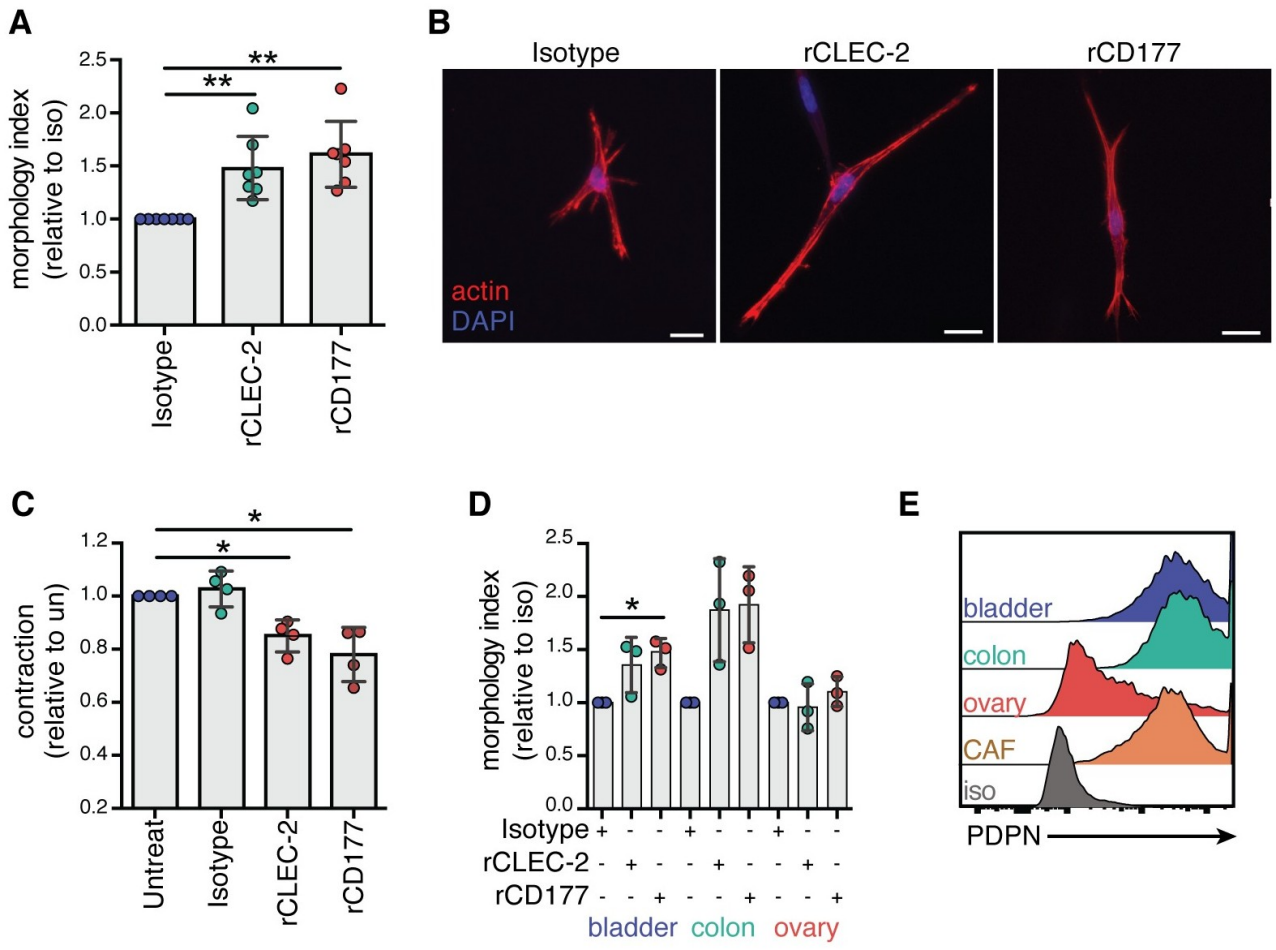
CD177 and CLEC-2 inhibit PDPN-mediated contractility in primary human CRC CAFs. **(A)** Graph showing morphology index of WT CAFs treated with isotype control, recombinant human CLEC-2, or recombinant human CD177. Dots represent the average of >50 cells in four fields of view per experiment, and six independent experiments. Data are plotted relative to the isotype control for each experiment. **(B)** Representative images of cells with various treatments in the 3D gels. Scale bar, 20 μm. **(C)** Contraction of WT CAFs treated with an isotype control, CLEC-2, or CD177 proteins relative with untreated CAFs. Dots represent the average of 2–3 wells per condition and graph comprises data from 4 independent experiments. **(D)** Morphology index of three different primary human fibroblasts from healthy tissues (bladder, colon, and ovary) treated with an isotype control, CLEC-2, or CD177 recombinant protein. Dots represent the average of 4 fields of view per experiment and graph represents data from three independent experiments. *p < 0.05, **p < 0.01, Kruskal-Wallis with Dunn’s multiple comparisons test. **(E)** Histogram showing representative staining for PDPN expression on the different fibroblasts compared with isotype control.

Next, we sought to study the functional outcome of CD177 binding to PDPN on CAFs. Notably, three dimensional morphological analyses showed that CD177 caused significant elongation of CRC CAFs, similarly to the known PDPN binding partner CLEC-2 (Fig 5A and 5B). In addition, both CLEC-2 and CD177 inhibited CAF contractility by approximately 20% (Fig 5C). These effects mimicked the phenotype that we observed in PDPN^-/-^ cells (Fig 4B), indicating that CD177 acts as an antagonist of PDPN functions in CAFs. Next, to confirm whether this phenotype was conserved across multiple types of fibroblasts, the effect of CD177 and CLEC-2 on primary human fibroblasts from healthy colon, bladder, and ovarian tissue were analyzed. Increased elongation was observed only in fibroblasts from the bladder and colon, not in those from the ovary (Fig 5D). Upon FACS analysis of the surface levels of PDPN in these cells, we found that bladder and colon fibroblasts expressed high levels of PDPN, on par with those from CAFs; in contrast, the majority of ovary fibroblasts expressed did not express PDPN (Fig 5E). This result indicates that CD177 and CLEC-2 function as general modulators of fibroblast elongation and contractility through PDPN engagement. Together, these data show that the novel binding partner CD177 is a functional antagonist of PDPN and increase our understanding of CLEC-2 functions in the human system, which has been mainly characterized in mice so far.

In summary, in this study we elucidate, to our knowledge for the first time, the cell surface interactome of an understudied cancer-associated protein, PDPN. We shed light on the biological processes modulated by PDPN in human fibroblasts, and the functional cellular outcomes that occur as a result of PDPN engagement by CLEC-2 and the novel binding partner CD177. Taken together, our findings suggest that interactions with immune cells could impinge on CAF functions in the tumor microenvironment in previously unappreciated ways.

## Discussion

Cell surface-expressed proteins and their interacting partners play a key role in sensing the microenvironment, acting as a signaling gateway that controls cell communication and cellular responses. In tumors, CAFs are increasingly recognized as key cellular players that critically influence and modulate tumor growth, metastasis, and anti-tumor immunity [1, 2, 34]. Despite representing emerging targets for drug development efforts, the markers and molecular underpinnings that dictate CAF functions remain in many cases ill-defined, especially regarding the molecules and pathways that dictate CAF-immune cells interactions. While the exact mechanisms through which CAFs influence tumor progression have been poorly characterized, these functions are importantly regulated by the receptor-ligand crosstalk at the interface between CAFs and other cells in the TME.

A multitude of studies have shown that PDPN expression is increased in cancer and frequently correlates with poor prognosis, suggesting an important role for this protein in cancer progression and/or immunoevasion, and pointing towards PDPN as a potential target for cancer therapeutics. Notwithstanding these observations, our basic knowledge of the functions of PDPN remain remarkably limited. Here, we sought to better understand basic aspects of PDPN function, particularly in the context of CAFs. First, we demonstrated that PDPN expressed by activated CAFs in the tumor is linked to poor prognosis. Additionally, we performed FACS analysis on primary tumor samples to show on the protein level that CAFs also are a major source of PDPN within the tumor microenvironment.

An important step towards understanding membrane protein function is the elucidation of its interactome, which initiate cell-cell interactions and often trigger cell signaling upon engagement of the cognate ligands. Despite this fact, interactions that take place on the cell surface are largely understudied due to technical challenges. We have developed a screening platform that overcomes several of these hurdles, enabling large-scale and high sensitivity identification of membrane protein interactions, which excels at capturing in trans (cell to cell) receptor-ligand interactions, often underrepresented by most screening technologies [7, 19]. To the best of our knowledge, this study presents the first unbiased assessment of PDPN binding partners on the cell surface, leading to identification of the neutrophil protein CD177 as a novel PDPN binder and thus suggesting a new molecular bridge between CAFs and neutrophils. At the molecular level we show that the interaction between CD177 and PDPN monomers is characterized by a micromolar binding affinity, indicating that this interaction may be particularly relevant at the interface between neutrophils and CAFs, where protein clustering may increase binding avidity and thus trigger a cellular response. It is also tempting to speculate that the novel interaction identified may be in part responsible for a yet uncharacterized interaction between CAFs and tumor-infiltrating Tregs, which have been shown to selectively express CD177 [29]. Further studies should certainly address these possibilities, as they may open new therapeutic avenues focused on regulation of highly immunosuppressive cell types such as Tregs.

Following validation of the CD177-PDPN interaction, we perform an in-depth proteomics analysis in primary human fibroblasts, where PDPN functions have not been studied in depth, despite their association with poor patient outcomes. This study reveals new molecular players that participate in signaling cascades previously assigned to PDPN, such as cell migration and cytoskeleton rearrangement, or cell adhesion. For example, PDPN stimulation resulted in increased phosphorylation of STAT3, a transcription factor involved in inflammation, tumorigenesis, and fibrosis [35–37]. It has recently been shown that CLEC-2 regulates microtubule-mediated deposition of extracellular matrix components by murine lymph node fibroblastic reticular cells (FRCs) in a mechanism that may involve PHLB1/2 (also known as LL5-β) [33]. Similarly, our results suggest that both CLEC-2 and the novel PDPN binder, CD177, induced rapid changes in the global phosphoproteome, including a marked decrease in phosphorylation of PHLB1/2. Together, this indicates that PDPN may signal through similar mechanisms in mouse and human fibroblasts, which will be interesting to explore in further studies.

Remarkably, this analysis also uncovered numerous proteins implicated in control of cell proliferation and survival that were unexpectedly and significantly hyper- or hypo-phosphorylated in response to CLEC-2 and CD177. As such, BCL9L and WWTR1/TAZ have been shown to play important roles in tumorigenesis through regulation of cell proliferation and apoptosis [38–40]. In addition, PDPN engagement led to differential phosphorylation of effectors known to play a role in inhibition of cell proliferation and angiogenesis, such as FILIP1L [41, 42], or ELAVL1, a well-described substrate of cyclin-dependent kinase 1 [43]. Previous studies have demonstrated a clear link between matrix stiffness, fibroblast contractility, and increased proliferation [44–46]; however our study is the first to report a role for PDPN in these processes, and moreover, it highlights cytosolic effectors that may mediate PDPN downstream signaling. Although comprehensive in nature, we cannot conclusively demonstrate that the changes observed in the phosphoproteome are exclusively mediated by PDPN expressed on the CAFs. This issue is fundamentally due to the amount of sample required for the global phospoproteome assessment, which has precluded us from performing similar experiments using PDPN-deficient primary CAFs. Notwithstanding this limitation, we successfully generated and functionally characterized PDPN KO CAF, which supported some of the findings from the proteomics study, such as an important role for PDPN in cell growth.

Collectively, our findings indicate that PDPN is crucial for maintaining key physiological properties and homeostasis of human CAFs leading to increased proliferation and differential signaling transduction, which ultimately supports tumor growth. As such, PDPN may directly enhance the pro-tumorigenic properties of CAFs. Furthermore, it’s possible that neutrophils and dendritic cells (the main cell types expressing CD177 and CLEC-2) may play previously unappreciated roles in modulating actomyosin contractility and overall CAF homeostasis. We hypothesize that in the TME, the high PDPN expression contributes to a highly contractile environment that acts as a barrier preventing T cell infiltration in the tumor bed. In addition, PDPN signaling sustains CAF proliferation, creating an environment favorable for tumor growth. In this scenario, increased CD177^+^ and CLEC-2^+^ immune cell infiltration could ameliorate PDPN functions, leading to reduced CAF contractility and possibly altering their activation state, ultimately hampering CAF-mediated enhancement of tumor growth.

Numerous clinical trials are aimed at relieving immunosuppression and enabling more immune cell infiltration into tumors, with a focus on cytotoxic T cells. In light of our results, it is tempting to speculate that modulation the PDPN/CLEC-2/CD177 axis may represent an alternative approach to remove obstacles for T cell entry into tumor beds, by lessening CAF contractility and ultimately decreasing tissue stiffness. While it is likely that in many tumors only low numbers of dendritic cells and neutrophils directly interact with the PDPN CAFs, new immunotherapies that engender more immune infiltration could shift the balance and lead to enough CLEC-2/CD177 in the tumor stroma to collectively inhibit PDPN and the pro-tumor CAF functions. Together, these results point towards a prominent role for CLEC-2 and CD177-expressing cells in the physiological and possibly immunosuppressive properties of CAFs, warranting further investigation of the consequences of these interactions *in vivo* and as possible avenues for new therapies.

In summary, starting from elucidation of the cell surface interactome of the cancer-associated protein PDPN, these data suggest unappreciated molecular interactions between stromal and immune cells that modulate the physiology of the CAFs, ultimately suggesting new avenues for therapeutic development.

## Supporting information

S1 File P-values from biomarker multivariate analysis (related to Fig 1). (DOCX)

S1 Fig PDPN^+^ CAFs are associated with poor survival outcomes in CRC patients. **(A, B)** Survival probability of patients from GSE39582 with stage II **(A)** or stage IV **(B)** disease, split into tertiles by PDPN expression levels. Throughout the figure, logrank p-values are associated with Kaplan-Meier curves while Cox proportional hazard (CoxPH) p-values are associated with the univariate models detailed in Table 2. **(C)** Example FACS plots from analysis of human CRC tumors to show gating strategy for major populations. **(D)** Major cell populations analyzed for PDPN expression as a percentage of total live cells. **(E)** PDPN MFI within all cell populations isolated from CRC tumors. **(F, G)** Survival probability of patients split into tertiles by levels of FAP^+^ fibroblast signature in GSE33113 (**F**) or GSE39582 (**G**). **(H, I)** Survival probability of patients with stage II disease from GSE39582 split into tertiles by levels of the activated fibroblast signature (**H**) or the FAP^+^ fibroblast signature (**I**).
(TIF)

S2 Fig CLEC-2 and CD177 have a profound effect on the CAF phosphoproteome. **(A)** Density plots depicting the changes observed in phosphorylated residues (x-axis) versus total protein abundance changes (y-axis), for the 70% protein overlapping proteins between both assays, in all four conditions. **(C)** Heatmap representing the protein phosphorylation fold change observed following CLEC-2 and CD177 stimulation, as shown in Fig 4B and 4C, including the identity of the phosphoproteins for which significant changes were observed.
(PNG)

S1 Table Summary statistics for the global phosphoproteome analysis. (XLSX)

S2 Table Results for the global protein analysis of WT CAFs treated with CD177 or CLEC-2. (XLSX)

S3 Table Results for the global protein analysis of WT CAFs treated with CD177 or CLEC-2. (XLSX)

S4 Table Gene ontology enrichement. Decription of the broad gene ontologies categories used to classify the global phosphoproteome changes reported in Fig 3D.
(XLSX)

S5 Table List of GO enrichment terms and the p-value for individual phosphosites. (XLSX)

S6 Table Classification of detailed GO ontology terms into broad categories. (XLSX)
