# Supplementary material for: The neutrophil protein CD177 is a novel PDPN receptor that regulates human cancer-associated fibroblast physiology
Source: PLoS One. 2021 Dec 8;16(12):e0260800. doi: 10.1371/journal.pone.0260800 (PMC8654239; doi:10.1371/journal.pone.0260800)
Supplement: S1 File — (DOCX) [file pone.0260800.s001.docx]

**S1 File. P-values from biomarker multivariate analysis (Related to Figure 1).**

**PDPN:** GSE33113: Adding CMS subtype to the model did not significantly improve the model fit (likelihood ratio test, p = 0.34).

**PDPN:** GSE39582 stage 2: adding CMS subtype did not improve the model fit (p = 0.59)

**PDPN:** GSE39582 stage 4: CMS subtype did not provide additional predictive power for RFS in stage IV either (likelihood ratio test, p = 0.87).

**Act fib and FAP+ fib signatures:** GSE33113: including CMS subtype did not further improve model fit (likelihood ratio tests, p = 0.239 and 0.099 respectively).

**Act fib and FAP+ fib signatures:** GSE39582 CMS subtype again did not improve model fit (p = 0.246 and 0.314 respectively).
