# Supplementary figures and images for: The neutrophil protein CD177 is a novel PDPN receptor that regulates human cancer-associated fibroblast physiology
Source: PLoS One. 2021 Dec 8;16(12):e0260800. doi: 10.1371/journal.pone.0260800 (PMC8654239; doi:10.1371/journal.pone.0260800)

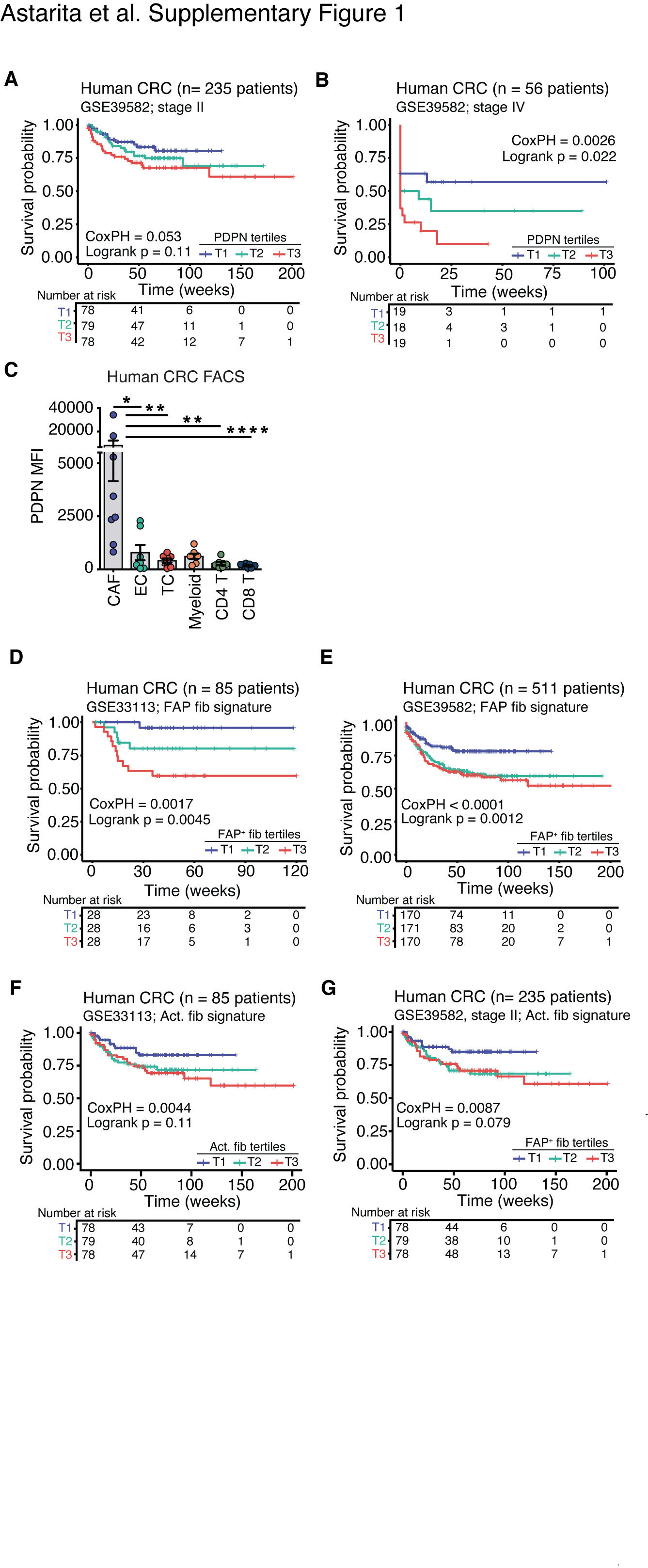

Supplement: S1 Fig — (A, B) Survival probability of patients from GSE39582 with stage II (A) or stage IV (B) disease, split into tertiles by PDPN expression levels. Throughout the figure, logrank p-values are associated with Kaplan-Meier curves while Cox proportional hazard (CoxPH) p-values are associated with the univariate models detailed in Table 2. (C) Example FACS plots from analysis of human CRC tumors to show gating strategy for major populations. (D) Major cell populations analyzed for PDPN expression as a percentage of total live cells. (E) PDPN MFI within all cell populations isolated from CRC tumors. (F, G) Survival probability of patients split into tertiles by levels of FAP+ fibroblast signature in GSE33113 (F) or GSE39582 (G). (H, I) Survival probability of patients with stage II disease from GSE39582 split into tertiles by levels of the activated fibroblast signature (H) or the FAP+ fibroblast signature (I). (TIF) [file pone.0260800.s002.tif]

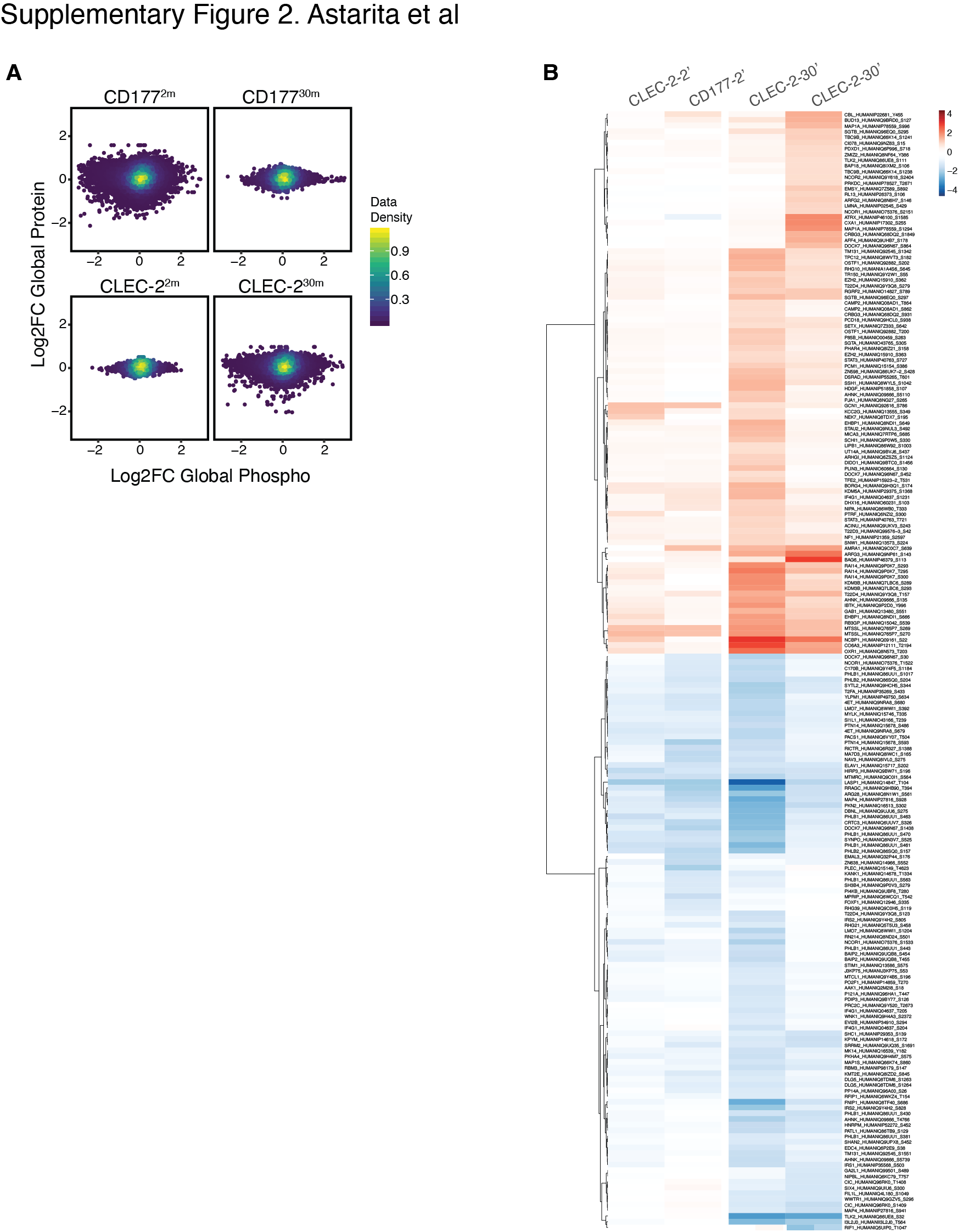

Supplement: S2 Fig — (A) Density plots depicting the changes observed in phosphorylated residues (x-axis) versus total protein abundance changes (y-axis), for the 70% protein overlapping proteins between both assays, in all four conditions. (C) Heatmap representing the protein phosphorylation fold change observed following CLEC-2 and CD177 stimulation, as shown in Fig 4B and 4C, including the identity of the phosphoproteins for which significant changes were observed. (PNG) [file pone.0260800.s003.png]
